# Adipose-derived Stem Cell Conditioned Media Extends Survival time of a mouse model of Amyotrophic Lateral Sclerosis

**DOI:** 10.1038/srep16953

**Published:** 2015-11-20

**Authors:** Christine V. Fontanilla, Huiying Gu, Qingpeng Liu, Timothy Z. Zhu, Changwei Zhou, Brian H. Johnstone, Keith L. March, Robert M. Pascuzzi, Martin R. Farlow, Yansheng Du

**Affiliations:** 1 Department of Neurology, Indiana University School of Medicine, Indianapolis, IN; 2 Department of Orthopedic Surgery, The 2nd Affiliated Hospital of Harbin Medical University, Harbin, P.R.C; 3 Department of Medicine, Indiana University School of Medicine, Indianapolis, IN.

## Abstract

Adipose stromal cells (ASC) secrete various trophic factors that assist in the protection of neurons in a variety of neuronal death models. In this study, we tested the effects of human ASC conditional medium (ASC-CM) in human amyotrophic lateral sclerosis (ALS) transgenic mouse model expressing mutant superoxide dismutase (SOD1^G93A^). Treating symptomatic SOD1^G93A^ mice with ASC-CM significantly increased post-onset survival time and lifespan. Moreover, SOD1^G93A^ mice given ASC-CM treatment showed high motor neuron counts, less activation of microglia and astrocytes at an early symptomatic stage in the spinal cords under immunohistochemical analysis. SOD1^G93A^ mice treated with ASC-CM for 7 days showed reduced levels of phosphorylated p38 (pp38) in the spinal cord, a mitogen-activated protein kinase that is involved in both inflammation and neuronal death. Additionally, the levels of α-II spectrin in spinal cords were also inhibited in SOD1^G93A^ mice treated with ASC-CM for 3 days. Interestingly, nerve growth factor (NGF), a neurotrophic factor found in ASC-CM, played a significant role in the protection of neurodegeneration inSOD1^G93A^ mouse. These results indicate that ASC-CM has the potential to develop into a novel and effective therapeutic treatment for ALS.

Amyotrophic lateral sclerosis (ALS), a progressive neurodegenerative disease, is defined by progressive loss of motor neurons, resulting in paralysis and ultimately death. The process is generally takes 2 to 5 years after diagnosis^1^. The degeneration of motor neurons in the spinal cord results in muscle atrophy and paralysis^2^. There are about two ALS incidences per 100,000 persons in the United States annually^3^. The majority cases are sporadic in origin and 5–10% are familial (FALS)^4^. Not only are both cases highly similar in clinical course, pathophysiology, and outcome, the mechanisms underlying disease progression are also posited to be the same^5^. 15–20% FALS are linked to mutant Zn/Cu superoxide dismutase^6^,^7^ (SOD1), which led to the creation of SOD1^G93A^ mouse model. SOD1^G93A^ mouse is currently the most extensively studied animal model of ALS^8^,^9^ that exhibit the major hallmarks of ALS, which include motor neuron pathology and progressive paralysis^8^,^9^. Researchers using this model has discovered an array of processes from ALS onset to end, including glutamate excitotoxicity, glial cell activation, oxidative damage, neuroinflammation, aberrant protein folding, mitochondrial dysfunction and axonal transport^4^,^10^.

Oxidative stress and motor neuron excitotoxic death have been linked to neuroinflammatory responses, for example, elevations of pro-inflammatory cytokines in the CNS^11^,^12^, astrocyte^13^ and microglia activation^14^. These pathogenic hallmarks are thought to play key roles in motor neuron death and ALS progression.

It has been reported that p38 mitogen activated protein kinase (MAPK) are involved in the motor neuron death^15^,^16^,^17^. Phosphorylated p38 MAPK was increased in the spinal cords of SOD1^G93A^ mice^15^,^16^,^18^ and human ALS patients^19^. Moreover, the inhibition of p38 MAPK pathway extended motor neuron survival and also decreased the activation of microglial in ALS mouse spinal cord^17^, this suggests that the pathogenic aspect of p38 MAPK pathway play a significant role in ALS motor neuron cell death and neuroinflammation.

Activity of the Ca^2+^-activated protease, calpain, has also been observed to be increased in the SOD1^G93A^ mouse^20^,^21^ due to increased levels of calcium in the cytosol caused by excitotoxicity^22^,^23^. Activated calpain cleaves cytoskeletal proteins, such as α-II spectrin and results in the accumulation of α-II spectrin and formation of inclusions located in motor neurons^20^. Inhibition of calpain via expression of its endogenous inhibitor, calpastatin, slowed degeneration of SOD1^G93A^ motor neurons^23^.

Since multiple pathways for neuronal death are involved in the development and maintenance of this disease, an effective multi-target approach may be required to treat ALS^10^. Additionally, ALS treatment can only be given after disease onset, because there are no specific predictors, clinical diagnosis, or biomarkers^4^. Lots of pharmacologic therapies used in both ALS clinical trials and stringent testing have been unsuccessful^24^. These failures at pre-clinical or clinical stage may attribute to the treatment’s inability to target more than one neuronal death pathway, resulting in an incomplete or ineffective blocking of motor neuron cell loss and ALS disease progression.

Adipose-derived stem cell conditioned media (ASC-CM), a biologically-derived reagent containing a multitude of neuroprotective and neurotrophic factors, such as brain-derived neurotropic factor (BDNF), nerve growth factor (NGF), vascular endothelial growth factor (VEGF), hepatocyte growth factor (HGF), and insulin-like growth factor-1 (IGF-1)^25^,^26^,^27^,^28^ was selected as ASC-CM has been previously shown to be neuroprotective by using both animal and cell culture models of neurodegeneration^27^,^29^,^30^. Our previous studies show that ASC-CM protected against hypoxia-ischemia-induced damage by blocking the activation of p38 MAPK^27^. NGF is a secreted growth factor in the central and peripheral nervous system. It is important in survival, growth and maintenance of specific types of neurons. The neurodegenerative disorders were due to the lack of hormones or growth factors^31^. NGF level was decreased in ALS dorsal spinal cord^32^. In this study, we evaluated the effectiveness of ASC-CM and investigated the role of NGF, a neurotrophic factor identified in the ASC-CM, in ASC-CM treatment for SOD1^G93A^ mouse.

## Materials and Methods

### Animals

SOD1^G93A^ heterozygous mice that overexpressing human SOD1-G93A mutation and wild type mice (WT) (all with B6SJL genetic background) were purchased form the Jackson Laboratory (Bar Harbor, ME, USA) and bred in the Animal Center of Indiana University School of Medicine. All animal procedures were performed in accordance with the protocols approved and authorized by the Institutional Animal Care and Use Committee at Indiana University School of Medicine. SOD1^G93A^ mice were identified by PCR performance with DNA derived from tail tissue using a protocol provided by The Jackson Laboratory^33^.

### Animal behavioral Assessment

As described in a previous paper^33^, behavioral assessment was performed beginning at 90 days of age. SOD1^G93A^ mice were randomly assigned to 5 different treatments of “ASC-CM”, “ASC-CM-NGF Ab”, “NGF Ab”, “NGF” or “vehicle”. SOD1^G93A^ mice were tested twice a week on a Rotarod apparatus at a speed of 15 rpm (ENV-575M; Med Associates, Inc., St. Albans, VT, USA) and up to 3 trials per day. Mice that were unable to remain on the Rotarod for 10 minutes were determined as disease onset, this performance was further tested the following day under same parameters to further verify disease onset. Animal that could not right itself in 20 seconds when gently rolled on its side indicated the end stage (surrogate death time point). Mice were checked every morning for mortality and morbidity, and every afternoon for the righting reaction.

### Poteomic analysis of the presence of neurotrophic factors in human ACS-CM

Human ASC secrete over 400 proteins into the medium during culture^34^. We detected interesting factors in ASC-CM using an antibody array (RayBio Human Growth Factor Array I, RayBiotech, GA)

### Quantification of NGF by ELISA

NGF level in ASC-CM was measured using Human NGF ELISA kits (Abcam, Cambridge, MA, USA), an enzyme-linked immunosorbent assay (ELISA) kit, according to the manufacturer’s instructions^35^.

### Animal Treatment

Human adipose-derived stem cell conditioned media (ASC-CM) was made as previously described^29^,^36^ and injected intraperitoneally (i.p.) at a volume of 200 μl once daily. To neutralize the activity of NGF, a polyclonal anti-NGF antibody (Abcam, Cambridge, MA, USA) was introduced and tested for its specificity. No cross-reactivity has been detected after using it against VEGF, GDNF, IGF1 and BDNF.1 μg/μl of the antibody was incubated with ASC-CM at 5 μg/ml overnight at 4 °C^28^,^37^. NGF antibody neutralized ASC-CM treated mice group (ASC-CM-NGF Ab) received 200 ul ASC-CM-NGF antibody once daily. Additionally, as controls, the NGF Ab group daily received 5 μg/ml NGF antibody in 200 μl Basal Media Eagle (BME), the NGF group daily had 200 pg/ml human NGF (R&D systems, Minneapolis, MN, USA) in 200 μl BME, and the vehicle group mice were injected with 200 μl BME. One daily dose of ASC-CM, NGF antibody neutralized ASC-CM, NGF antibody, NGF or BME was given until a humane death endpoint for survival studies, and 3 or 7 days after onset for biochemical and immunohistochemical studies.

### Western Blot

Western blot analysis was performed with tissue lysates as previously described^33^,^38^ after 3 or 7 days of ASC-CM or BME treatment. Radio-immunoprecipitation assay (RIPA) buffer and protease inhibitor (Roche Diagnostics Corp., Indianapolis, IN, USA) was used to homogenize spinal cord samples. 15 μg/well of protein was loaded onto a 4–12% Bis-Tris gel, electrophoresed, then transferred to nitrocellulose membrane (Hybond N; Amersham Biosciences, Piscataway, NJ, USA). Blots were probed with rabbit polyclonal primary antibody against pp38 (1:1000; Millipore, Temecula, CA, USA), mouse monoclonal antibody against glial fibrillary acidic protein (1:1000, GFAP; Millipore), rabbit polyclonal antibody against ionized calcium binding adaptor molecule 1 (1:1000, Iba-1; Wako Chemicals USA, Inc., Richmond, VA, USA), and goat polyclonal antibody against α-II spectrin (1:200, Santa Cruz Biotechnology Inc., Santa Cruz, CA, USA) followed by horseradish peroxidase-conjugated secondary antibody (Santa Cruz Biotechnology Inc., Santa Cruz, CA, USA). Finally, visualized through utilizing enhanced chemiluminescence (Amersham Biosciences, Piscataway, NJ, USA), band intensities were quantitated by densitometric analysis (ImageJ, http://rsbweb.nih.gov/ij/).

### Immunohistochemistry

Mice were anesthetized and perfusion-fixed with 4% paraformaldehyde (PFA) after treatments with ASC-CM or BME. PFA-fixed and paraffin embedded spinal cords were sectioned (15 μm) serially via the lumber enlargement and immunostained with anti-microtubule-associated protein 2 antibody (1:1000, MAP2; Millipore), then followed by a biotinylated anti-mouse IgG antibody (Sigma-Aldrich Corp.). Sections were visualized under the microscope after applying 3,3′-diaminobenzidine (DAB) substrate solution. A 40x objective light microscope (Nikon, Japan) was used to count large motor neurons in the ventral horn of the lumbar spinal cord^39^,^40^. 6–8 sections of each lumbar were counted. Data was reported as number of motor neurons per section as previously described^33^,^41^.

### Statistical Analysis

One-way analysis of variance (ANOVA) was used for statistical analyses and comparisons for the differences between groups. All data are expressed as mean ± standard error of the mean (SEM). Differences between two means were considered significant when p was equal or less than 0.05.

## Results

### Neurotrophic factors in ASC-CM

We tested interesting neurotrophic factors in ASC-CM by using a Human Growth Factor Antibody Array (containing 41 growth factor antibodies). Image showed the presence of NGF, VEGF, GDNF, IGF-1 in human ASC-CM. BDNF was detected in human ASC-CM by using enzyme-linked immunosorbent assay (ELISA) kit (Chemicon, Temecula, CA, USA)^27^.

### ASC-CM significantly reduced disease progression and increased life span of SOD1^G93A^ mice, while NGF antibody attenuated this effect

**Figure 1:**
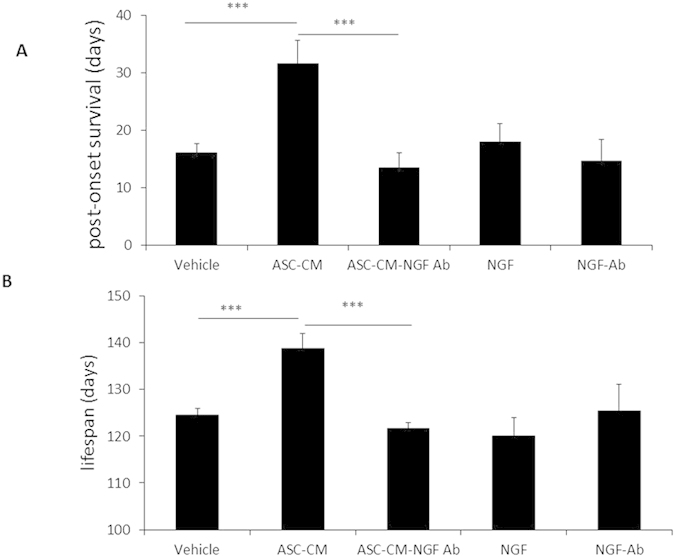
Treatment with ASC-CM extends post-onset survival and lifespan of SOD1^G93A^ mice. Symptomatic SOD1^G93A^ mice were given daily ASC-CM, NGF depleted ASC-CM treatment or vehicle until endpoint. Duration of disease is reported as days of post-onset survival and measured by days between disease onset and humane death endpoint. Lifespan is the number of days from birth to the humane death endpoint. Symptomatic mice treated with ASC-CM daily (n = 5) had a significant increased survival time as compared to vehicle treated SOD1^G93A^ mice (n = 7). The averages of post-onset survival time were significantly increased in mice given ASC-CM compared to vehicle. ASC-CM-treated mice with disease onset (n = 5) had a significant lifespan extension when compared to vehicle treated SOD1^G93A^ mice (n = 7), but NGF antibody neutralized ASC-CM, NGF antibody, or NGF did not affect post-onset survival times (n = 5) (**A**). Increased post-onset survival time of ASC-CM treated mice was responsible for the significantly extended lifespan relative to vehicle mice. However, NGF antibody attenuated this effect in ALS mice. As controls, NGF antibody or NGF did not affect lifespans of mice (**B**). Statistical analysis was performed by using one-way ANOVA. ***p < 0.005 vs. vehicle.

SOD1^G93A^ mice were treated with ASC-CM from disease onset to a humane death endpoint. The duration from disease onset to death was measured. Data was reported as number of days of post-onset survival (Fig. 1A). The SOD1^G93A^ mice given ASC-CM had increased post-onset survival times (31.6 ± 4.1 days, n = 5; ***p < 0.005) as compared to vehicle-treated mice (16.1 ± 1.6 days, n = 7). Interestingly, NGF antibody neutralized ASC-CM didn’t affect post-onset survival times (13.4 ± 2.7 days, n = 5) as compared to vehicle-treated mice. ASC-CM treated mice also had significantly longer lifespans (138.8 ± 3.1 days, n = 5, Fig. 1B) versus the vehicle counterparts (124.4 ± 1.5 days, n = 7) and as expected, NGF antibody attenuated this effect (121.7 ± 1.2, n = 5). As controls, we treated NGF Ab group mice and NGF group mice with 5 μg/ml NGF antibody and 200 pg/ml NGF, respectively and did not observe any effects of the NGF antibody and NGF on post-onset survival (14.6 ± 3.8 days, 18.0 ± 3.2 days vs. 16.1 ± 1.6 days) and lifespans (125.3 ± 5.8 days, 120.1 ± 3.9 days vs. 124.4 ± 1.5 days).

### ASC-CM increased the number of motor neurons in the lumbar spinal cord

**Figure 2:**
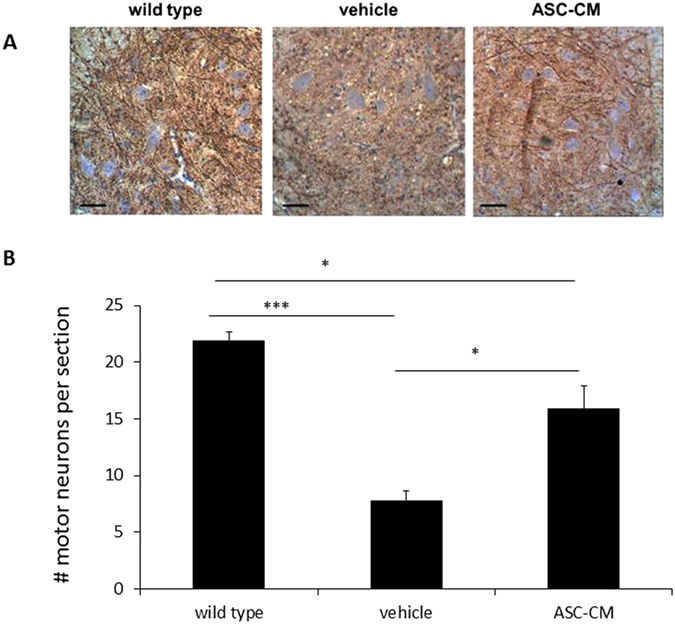
ASC-CM 7-day treatment of SOD1^G93A^ mice with disease showed increase number of motor neurons in the lumbar spinal. SOD1^G93A^ mice were given ASC-CM or vehicle for 7 days after disease onset. Motor neurons in the spinal cord area were identified by immunoreactivity to MAP2 antibody. Representative images showed an increased number of MAP2-positive neurons in lumbar spinal cords of SOD1^G93A^ mice treated with ASC-CM compared to vehicle. Scale bars: 100 μm. (**A**). MAP2 immunoreactive images covering the entire cross-sectional area from6–8 lumbar spinal regions for each mouse were quantitated. With ASC-CM treatment, a higher number of motor neurons was observed in SOD1^G93A^ mice with disease onset (n = 5) versus vehicle (n = 5). Wild type control data were generated from 4 mice (**B**). Experimental group data are presented as averages (±SEM) and statistical analyses were performed using one-way ANOVA. *p < 0.05; ***p < 0.005.

A positive correlation was observed between extended survival time of ASC-CM treated SOD1^G93A^ mice and the prevention of motor neuron loss. Changes in motor neuron number at 7 days following onset were analyzed in cross-sections of the lumbar spinal cords of SOD1^G93A^ mice (Fig. 2A). BME-treated control mice showed a marked (***p < 0.005) loss of motor neurons in the ventral horn of the lumbar spinal cord versus WT control (7.8 ± 0.84 and 21.9 ± 0.79, respectively; Fig. 2B), but this loss was inhibited by ASC-CM treatment (15.9 ± 2.0, p < 0.01 vs. vehicle). Although there was still significant motor neuron loss (p < 0.05) compared to age-matched and wild type mice, this loss was not as severe in the vehicle group (Fig. 2B).

### Administration of ASC-CM had no effect on mouse SOD1^wild type^ and mutant human SOD1^G93A^ expression in SOD1^G93A^ mice spinal cords

**Figure 3:**
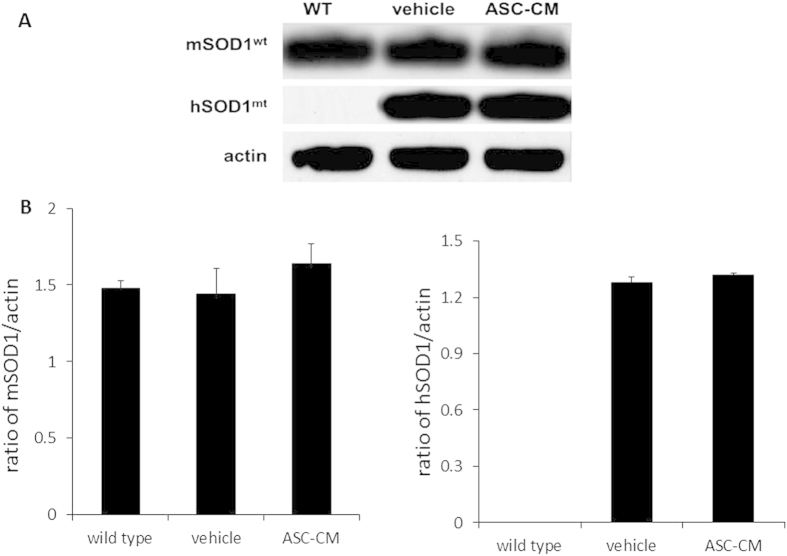
Treatment with ASC-CM did not affect expression of endogenous mouse wild type SOD1 and mutated human SOD1 transgene in spinal cord. Wild type control (WT) and SOD1^G93A^ mice (vehicle and ASC-CM) spinal cords were prepared as detailed in materials and methods. Levels of actin were unchanged and used as an internal loading control for endogenous mouse wild type SOD1 (mSOD1^WT^). There was no difference in mSOD1^WT^ levels, which was the loading control for mutated human SOD1 (hSOD1^G93A^). After 7 days of ASC-CM treatment, there was no change in expression of either endogenous mSOD1^WT^ or hSOD1^G93A^ mice in symptomatic SOD1^G93A^ mice. n = 3 per group.

Studies have shown that the transgene copy number (copies of the transgene with the G → A at position 93, human mutant form of SOD1) affects survival of the SOD1^G93A^ mouse^9^. In order to determine if ASC-CM has an effect on SOD1 gene expression, immunoblot analyses were performed on spinal cord homogenates after 7 days of ASC-CM treatment (Fig. 3A). Band density measurements showed that levels of both wild type endogenous mouse SOD1 (mSOD1^WT^) and mutated human SOD1 (hSOD1^G93A^) exhibited no differences with or without 7-day ASC-CM treatment (Fig. 3B). “Wild type/WT” mice express wild type endogenous mSOD1^WT^ but does not carry the mutated hSOD1^G93A^ transgene; “vehicle” and “ASC-CM” mice are the experimentally-treated SOD1^G93A^ transgenic mice that express wild type endogenous mouse SOD1 (mSOD1^WT^) in addition to the hSOD1^G93A^ transgene. Actin levels remain unchanged and assayed as loading controls for endogenous mSOD1^WT^. There were differences in endogenous mSOD1^WT^ levels, which then served as an internal control for mutated hSOD1^G93A^ expression.

### Daily ASC-CM treatment reduced p38 MAP kinase phosphorylation in SOD1^G93A^ mice spinal cords

**Figure 4:**
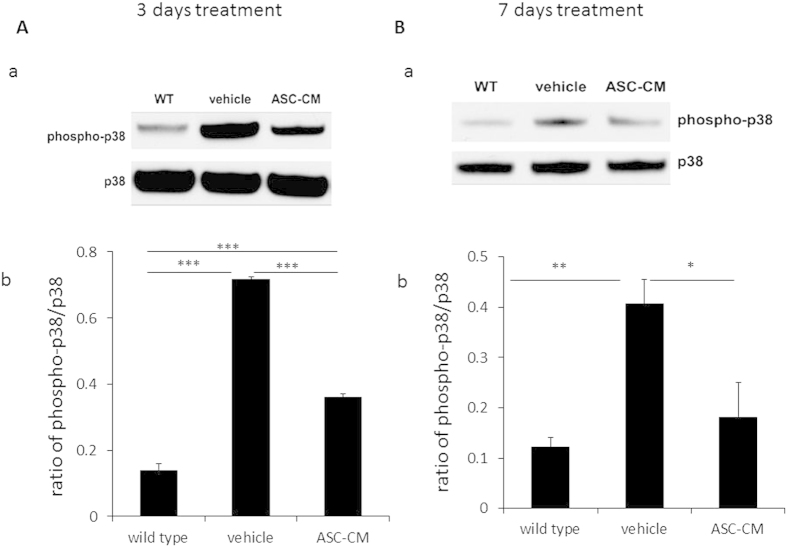
pp38 reduction was observed after 3 or 7 days of ASC-CM treatment in SOD1^G93A^ mice spinal cords after disease onset. SOD1^G93A^ mice with onset received ASC-CM or vehicle for 3 days or 7 days, while spinal cords were removed for Western blot. (**A**): (a) Representative immuoblots and (b) quantitation of band density showed reduced pp38 expression in SOD1^G93A^ mice spinal cords after 3 days ASC-CM treatment versus BME treatment. (**B**): (a) Representative immunoblots and (b) densitometric quantitation demonstrated reduced pp38 expression in 7 days ASC-CM-treated mice compared to BME-treated mice. Unchanged total p38 levels were used as internal loading controls. Values are shown as mean density ± SEM and statistical analyses were performed using one-way ANOVA. *p < 0.05; **p < 0.01; ***p < 0.005; n = 3 per group.

Phosphorylated p38 (pp38) levels in spinal cords were examined at 3 days (Fig. 4A) and 7 days (Fig. 4B) following disease onset with or without ASC-CM administration during that time period. In agreement with other reports using the SOD1^G93A^ mouse^17^,^19^, p38 phosphorylation was elevated in SOD1^G93A^ mice after disease onset at both 3 days (0.72 ± 0.008, p < 0.005 vs. wild type and ASC-CM; Fig. 4A) and 7 days post-onset (0.41 ± 0.05, p < 0.01 vs. wild type; Fig.4B). pp38 levels in spinal cords were significantly decreased with ASC-CM treatment for 3 days (0.36 ± 0.01, p < 0.005 vs. vehicle and wild type; Fig. 4A) and for 7 days (0.18 ± 0.07, p < 0.05 vs. vehicle; Fig.4B) as compared to the vehicle groups. Levels of pp38 in ASC-CM treated animals were higher than WT controls at 3 days (0.14 + 0.02, p < 0.005 vs. ASC-CM group; Fig.4A).

### GFAP expression was reduced in the spinal cords of symptomatic SOD1^G93A^ mice treated with ASC-CM

**Figure 5:**
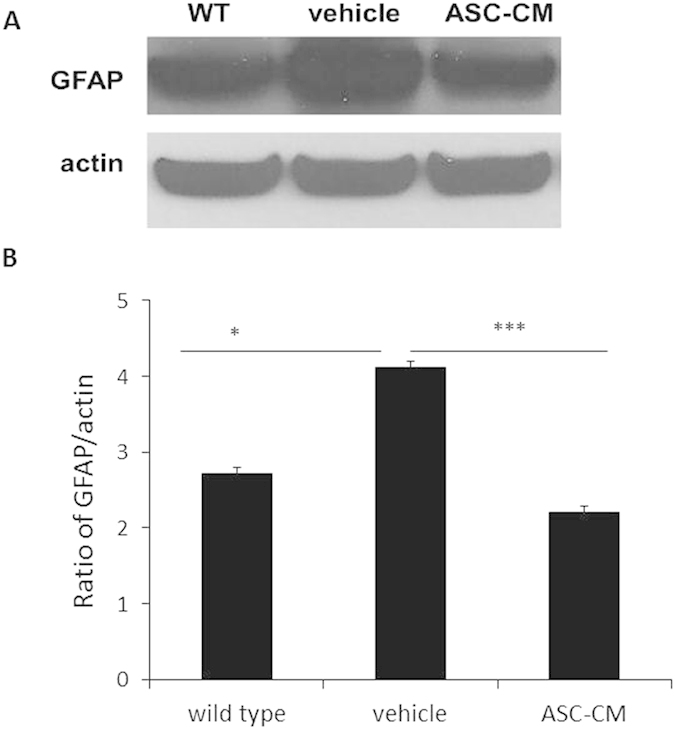
3-day treatment with ASC-CM showed reduced GFAP expression in the spinal cords of SOD1^G93A^ mice after disease onset. Figure SOD1^G93A^ mice with disease onset were given ASC-CM or vehicle for 3 days. Spinal cords were removed for immunoblot. Actin expression levels remained constant in all groups and were used as internal loading controls. (**A**) Representative immunoblots and (**B**) band quantitation demonstrated a decrease in spinal cord GFAP expression after 3-day ASC-CM treatment as compared to vehicle. Experiments were completed in triplicate with n = 3 per group. *p < 0.05; ***p < 0.005.

It has been demonstrated that motor neuron loss occurs partly through activated inflammatory cells in the surrounding area^42^. Because treatment with ASC-CM showed significant neuroprotection of motor neurons possibly via anti-inflammatory mechanisms thus far, glial activation after treatment with ASC-CM was examined to further explore this possibility. Expression of GFAP was measured in the spinal cords of SOD1^G93A^ mice after 3 days of treatment with ASC-CM or vehicle. Representative immunoblots demonstrated that there is significantly reduced GFAP expression with ASC-CM treatment (2.71 ± 0.08) versus vehicle (4.12 ± 0.07; ***p < 0.005; Fig. 5). The GFAP levels observed in the ASC-CM treated group were comparable to that of the wild type controls (2.20 ± 0.08).

### Administration of ASC-CM to SOD1^G93A^ mice with disease onset decreased CD11b expression in spinal cords

**Figure 6:**
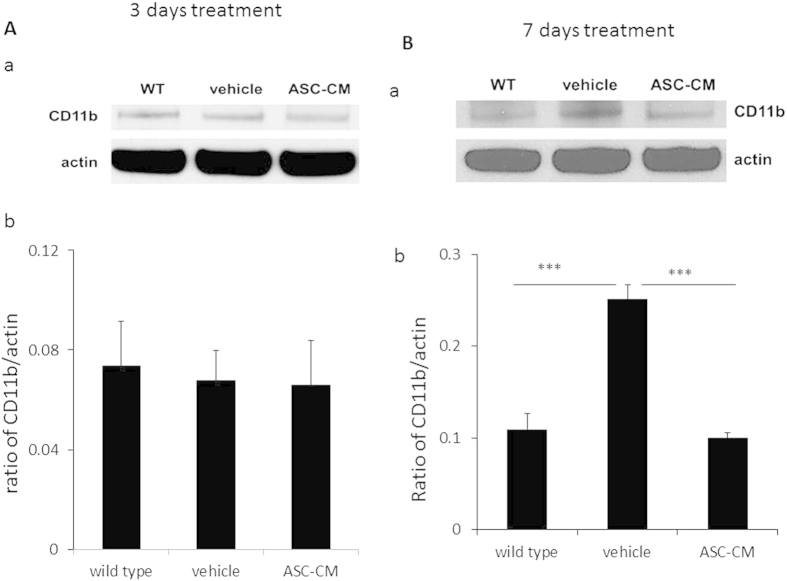
CD11b expression in spinal cords of symptomatic SOD1^G93A^ mice after 3 days or 7 days of ASC-CM treatment. SOD1^G93A^ mice were given 3 days or 7 days of ASC-CM treatment or vehicle upon disease onset. Spinal cords were processed Western blot as described in Experimental Procedures. Unchanged actin levels in all groups were used as internal loading controls. (**A**) (a) The representative immunoblots and (b) band densities that were measured showed no change in spinal cord CD11b expression after 7 days of ASC-CM treatment. (**B**) (a) The representative immunoblots and (b) band quantitation demonstrated a decrease in spinal cord CD11b expression after 7 days of ASC-CM treatment as compared to vehicle. n = 3, ***p < 0.005.

In order to further investigate glial activation after ASC-CM treatment, spinal cords were prepared for Western blot and examined for expression of CD11b, a glial marker found to be induced by neuroinflammation^14^. CD11b expression was observed at 3 days (Fig. 6A) and 7 days (Fig. 6B) after ASC-CM or vehicle treatment. 3 days of ASC-CM treatment demonstrated no differences in CD11b expression in all groups (group averages ± SEM: 0.073 ± 0.018 for wild type; 0.068 ± 0.012 for vehicle; 0.066 ± 0.018 for ASC-CM, Fig. 6A). However, 7 days of ASC-CM treatment demonstrated a significant decrease in CD11b expression in the spinal cord of ASC-CM-treated animals (0.109 ± 0.017, Fig. 6B)when compared to vehicle mice (0.251 ± 0.015 for vehicle, ***p < 0.005 versus wild type and ASC-CM), whose CD11b expression level was at a similar level to wild type mice (0.100 ± 0.005) after 7 days.

### ASC-CM treatment of symptomatic SOD1^G93A^ mice resulted in decreased expression of α-II spectrin in spinal cords

**Figure 7:**
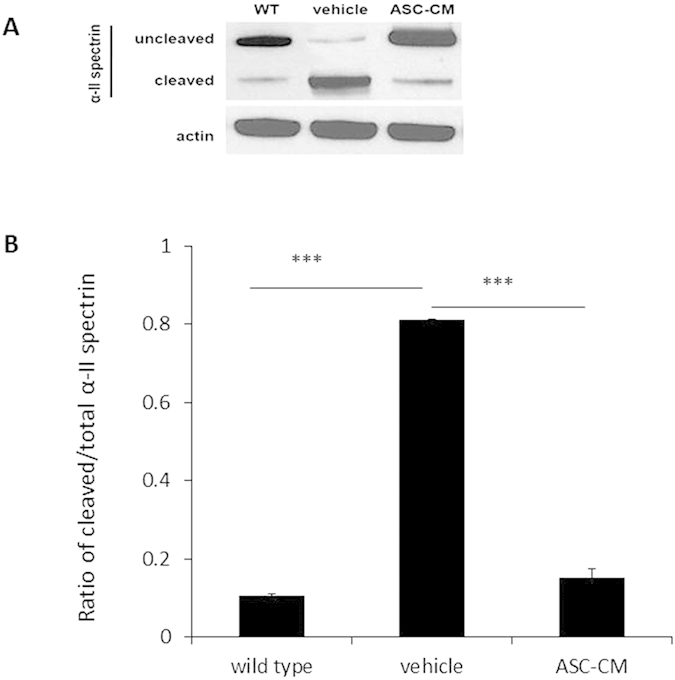
ASC-CM treatment for 3 days decreased α-II spectrin cleavage in symptomatic SOD1^G93A^ mice. SOD1^G93A^ mice were given ASC-CM or vehicle for 3 days. Western blot analysis was done to measure α-II spectrin. Unchanged actin levels expression were used as internal loading controls. (**A**) Representative immunoblots and (**B**) quantitation of band densities revealed a decrease in cleaved α-II spectrin expression at 3 days post-onset when ASC-CM treatment was given. Wild type, n = 5; vehicle, n = 3; ASC-CM, n = 4. ***p < 0.005.

ASC-CM’s effect on the calpain system in SOD1^G93A^ mice was determined by measuring expression of α-II spectrin, a known cleavage target of calpain^43^. After 3 days following disease onset, activity of the Ca^2+^-activated protease, calpain, was increased in SOD1^G93A^ mice spinal cords given vehicle(0.81 ± 0.002, n = 4, ***p < 0.005) as compared to wild type controls (0.10 ± 0.006, n = 3), consistent to a previous report^20^. Cleaved α-II spectrin was significantly decreased in SOD1^G93A^ mice receiving ASC-CM treatment as compared to mice given vehicle (0.15 ± 0.026, n = 3, ***p < 0.005, Fig. 7). Levels of actin remained unchanged for all groups and were used as internal loading controls.

## Discussion

This study was designed to confirm the effects of a biologically-derived reagent on an ALS animal model that is clinically relevant such that treatment was applied after disease onset was established in the widely used SOD1^G93A^ mouse model^8^. The data collected from this study demonstrate the effectiveness of ASC-CM treatment in prolonging post-onset survival and extending the lifespan of symptomatic SOD1^G93A^ mouse. These results are in agreement with previous experiments from our laboratory, showing that the treatment with ASC-CM is beneficial and protective of neurons *in vivo* during neuronal injury or challenge, such as in the hypoxic-ischemic rat neonatal brain^27^. The findings from this study and the previous *in vivo* studies suggest that ASC-CM has the potential as a therapeutic for neuronal degeneration.

Although the etiology of sporadic ALS is currently unknown, through the use of animal models and correlation with neuropathological findings from human ALS patients, several key mechanisms of neuronal death have been proposed to disrupt normal motor neuron function and lead to their degeneration. Current studies implicate glutamate-induced excitotoxicity, mitochondrial dysfunction, oxidative stress, neuroinflammation, aberrant axonal transport systems, endoplasmic reticulum (ER) stress, inhibition of proteosomal function, compromised blood-brain barrier, and abnormal protein aggregation^44^. There is much debate as to which processes contribute towards disease etiology and which events are secondary to the established disease state. This presents ALS drug development with difficulties as neuropathology-specific targeting of treatment may be ineffective.

Despite the mechanistic uncertainties underlying events leading to ALS neuropathologic changes, treatments like ASC-CM may prove to be beneficial as they are able to block or modify several neuronal death pathways due to the heterogeneity in their composition. ASC-CM has been found to contain a multitude of neurotrophic factors that could be beneficial for the diseased motor neuron^27^. It was shown that neurotrophic factors may play a critical role in protecting or delaying motor neuronal death in ALS mice^45^. In this study, neurotrophic factors in ASC-CM could access affected neurons in CNS by either directly crossing the blood brain barrier^46^,^47^ or going through the retrograde transport mechanism^45^,^46^. Additionally, since motor neuronal death in ALS mice showed a distal–proximal gradient, it is also possible some neurotrophic factors in ASC-CM increased lifespan of ALS mice by directly protecting the neuromuscular junctions in peripheral^45^. Furthermore, in order to overcome the short half-life of polypeptides in ASC-CM peripherally, we i.p. injected ASC-CM daily until the end of experiments^48^,^49^. Here, after post-onset treatment with ASC-CM peripherally, symptomatic SOD1^G93A^ mice have significant prolongation of post-onset survival times, which translated into an overall extension of lifespan. However, since many neurotrophic factors existing in ASC-CM may exert neuroprotective effects together as a neuroprotective mixture body^27^, deletion of single factor may fully remove its neuroprotective effects. In this study, we test this hypothesis by targeting the NGF contained in ASC-CM^50^. As predicted, NGF neutralized ASC-CM did not elongate the post-onset survival times and lifespan of SOD1^G93A^ mice.

Additionally, the lengthening of post-onset lifespan following ASC-CM administration was correlated with markedly increased numbers of motor neuron survival in the spinal cord lumbar area. Further examinations at the cellular level determined that the increase in motor neuron survival after ASC-CM treatment was accompanied by decreased expression of glial activation markers and phosphorylated p38 MAP kinase, which are important components of the neuroinflammation pathway^51^ contributing to neuronal death in the spinal cords of SOD1^G93A^ mice^17^. Other data from this study shows lower levels of cleaved α-II spectrin, which is a known substrate of both calpain and caspase-3^52^,^53^. Decreased cleavage of α-II spectrin in the spinal cords of ASC-CM-treated SOD1^G93A^ mice may indicate that calpain has protective effects against glutamate-induced neuronal apoptosis^20^,^21^,^23^.

Growing evidence has shown that NGF induces neuronal survival with low-affinity binding to the p75 neurotrophin receptor (p75NTR)^54^. Additionally, it was shown that NGF-induced neuroprotection against OGD insult by inhibiting OGD-induced p38 activation^55^ and against UV neurotoxicity by inhibiting calpain activity^56^. Furthermore, increased production of NGF in central nervous system (CNS) during diseases is able to suppress inflammation by switching the immune response to an anti-inflammatory^57^. Therefore, it is quite possible that NGF induced neuroprotection in ASC-CM-treated SOD1G93A mice via neuroprotection, inhibition of p38 and calpain, as well as anti-inflammation. It would be interesting to investigate by using appropriate models how NGF protects motor neurons in ASC-CM-treated SOD1^G93A^ mice and whether glial activation or inflammatory processes play important roles in this neuroprotective process.

Taken together, this study demonstrated that ASC-CM provides significant neuroprotection in the SOD1^G93A^ mouse model of ALS and extendsSOD1^G93A^ mice life span. NGF in ASC-CM plays a significant role in this neuroprotection. This protective ability may associate with the preservation of motor neurons and inflammatory pathway inhibition in the spinal cord. This study establishes the therapeutic potential of this agent for treating ALS. Additionally, ASC-CM also holds great promise as potential therapies for many other neurodegenerative diseases due to the validated beneficial effects in many experimental models.

## Additional Information

**How to cite this article**: Fontanilla, C. V. *et al*. Adipose-derived Stem Cell Conditioned Media Extends Survival time of a mouse model of Amyotrophic Lateral Sclerosis. *Sci. Rep*. **5**, 16953; doi: 10.1038/srep16953 (2015).
