# Corrigendum: Adipose-derived Stem Cell Conditioned Media Extends Survival time of a mouse model of Amyotrophic Lateral Sclerosis

**DOI:** 10.1038/srep20747

**Published:** 2016-04-15

**Authors:** Christine V. Fontanilla, Huiying Gu, Qingpeng Liu, Timothy Z. Zhu, Changwei Zhou, Brian H. Johnstone, Keith L. March, Robert M. Pascuzzi, Martin R. Farlow, Yansheng Du

Scientific Reports 5: Article number: 16953; 10.1038/srep16953 published online: 11202015; updated: 04152016.

In this Article, an additional affiliation for Keith L. March was omitted. The correct affiliation is listed below:

Krannert Institute of Cardiology and the Indianapolis VA Center for regenerative medicine

